# Genetics of MASLD: a diabetes perspective

**DOI:** 10.1007/s00125-026-06759-6

**Published:** 2026-06-08

**Authors:** Josh Bilson, Hanieh Yaghootkar

**Affiliations:** 1 School of Human Development and Health, Faculty of Medicine, University of Southampton, Southampton, UK; 2 School of Electronics and Computer Science, Faculty of Engineering and Physical Science, University of Southampton, Southampton, UK

**Keywords:** Genetics, Liver fat, MASLD, Metabolic dysfunction-associated steatotic liver disease, Review, Type 2 diabetes

## Abstract

Metabolic dysfunction-associated steatotic liver disease (MASLD) is highly prevalent in people with type 2 diabetes and represents a major contributor to liver-related and extrahepatic morbidity and mortality. Despite this strong epidemiological overlap, the clinical course of MASLD in diabetes is highly heterogeneous and not fully explained by conventional metabolic risk factors. Human genetic studies have provided important insights into this variability by revealing that hepatic steatosis and its downstream consequences can arise through biologically distinct pathways. Genome-wide association studies have identified multiple genetic loci influencing liver fat accumulation, disease severity and progression. These loci implicate diverse mechanisms, including liver-intrinsic defects in lipid handling, enhanced hepatic lipogenesis, and systemic metabolic dysfunction related to insulin resistance and adipose tissue biology. Genetic evidence indicates that similar degrees of hepatic steatosis may therefore reflect different underlying biological processes, with differing implications for glycaemic management, cardiovascular risk and liver disease progression. In this review, we examine the genetic architecture of hepatic steatosis and MASLD in the context of diabetes. We discuss evidence from family studies, genome-wide association analyses, imaging genetics and Mendelian randomisation, with an emphasis on cautious causal interpretation. We also explore gene–diabetes and gene–environment interactions that modify disease expression and may contribute to variability in clinical outcomes and treatment response. Finally, we consider the translational implications of MASLD genetics, including risk stratification, therapeutic target discovery and emerging genotype-informed approaches to clinical management. We highlight key research gaps, particularly the need for ancestry-diverse studies, improved phenotyping in diabetes populations, and integration of genetic analyses into prospective clinical trials. Together, current genetic evidence supports a mechanism-based framework for understanding MASLD heterogeneity in diabetes and provides a foundation for more precise approaches to risk assessment and management.

**Graphical Abstract:**

**Figure:**
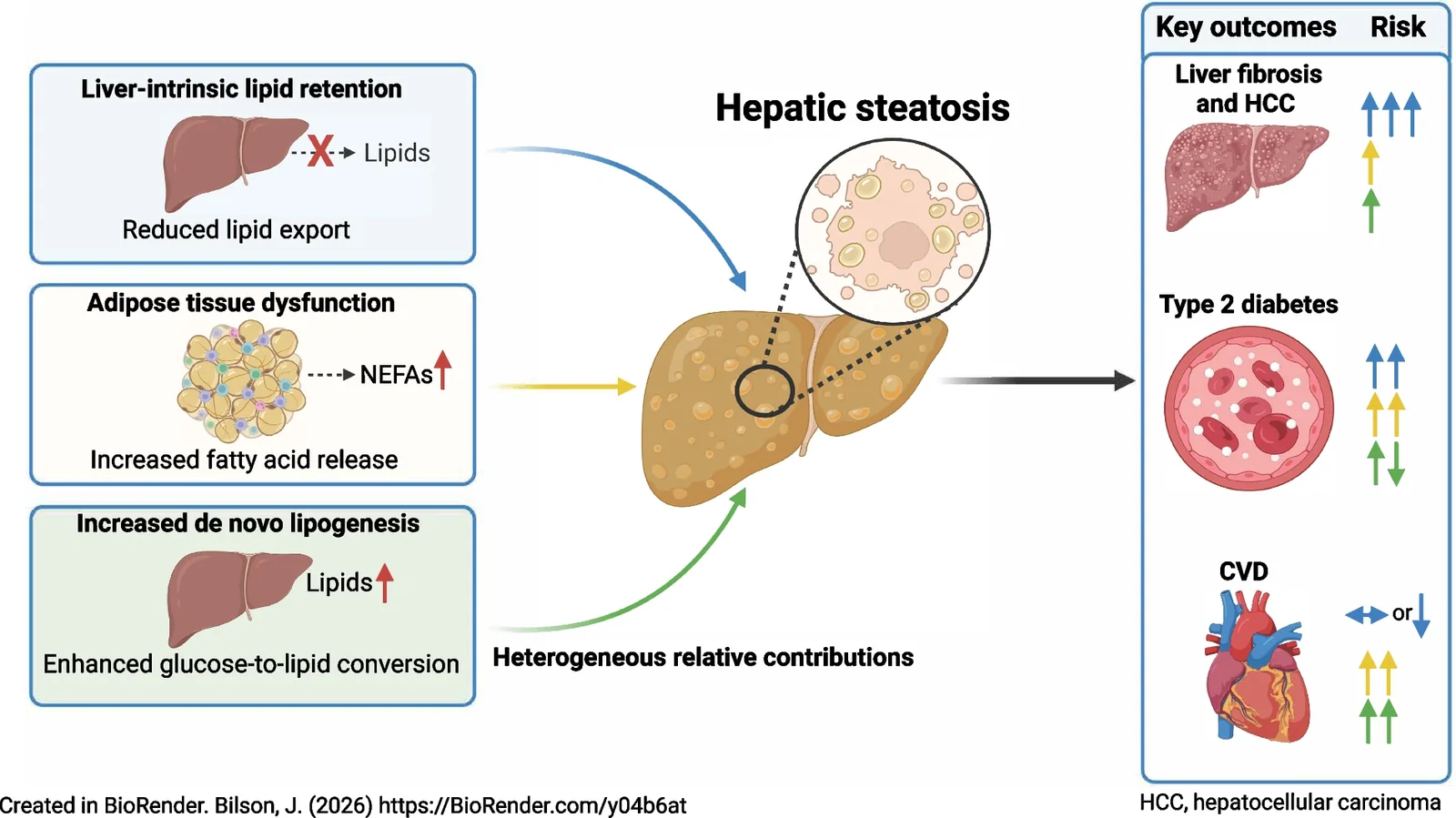

**Supplementary Information:**

The online version contains a slideset of the figures for download available at https://doi.org/10.1007/s00125-026-06759-6.

## Introduction

Metabolic dysfunction-associated steatotic liver disease (MASLD) is highly prevalent in people with type 2 diabetes, affecting approximately 60–75% of this population [1]. This large epidemiological overlap creates challenges for diagnosis, prognosis and treatment. MASLD can progress to fibrosis, cirrhosis and hepatocellular carcinoma and is also associated with increased risks of cardiovascular disease and chronic kidney disease [2]. The term ‘MASLD’ was introduced in June 2023 and is now widely recognised as the new name for non-alcoholic fatty liver disease (NAFLD) [3]. While there are differences in the diagnostic criteria between NAFLD and MASLD, there is almost a complete overlap between populations previously diagnosed with NAFLD and those now classified with having MASLD [4]. In this review, we use the term ‘MASLD’ throughout for consistency, recognising that much of the existing genetic evidence is derived from studies conducted under the previous NAFLD definition.

Despite the strong association between MASLD and type 2 diabetes, the clinical course of MASLD in people with diabetes is highly variable. Some individuals with long-standing diabetes and obesity have relatively mild liver disease, whereas others develop advanced fibrosis or hepatocellular carcinoma despite having comparable metabolic profiles [2]. Conventional risk factors such as BMI, glucose concentrations and lifestyle do not fully explain this heterogeneity, suggesting that inherited susceptibility contributes to disease expression.

Human genetics provides a framework to investigate this variability. Genetic approaches can help assess directionality between traits, for example whether liver fat accumulation contributes to insulin resistance or whether insulin resistance primarily promotes steatosis. Genetic studies have also shown that hepatic steatosis can arise through biologically distinct pathways, including liver-intrinsic defects in lipid handling and systemic metabolic processes related to insulin resistance and adiposity [5, 6]. These observations may explain why MASLD does not always align with the severity of the metabolic syndrome. Finally, genetic studies can identify risk loci with different functional consequences, offering insight into disease mechanisms and potential avenues for risk stratification and therapy.

In this review, we examine the genetic architecture of hepatic steatosis and MASLD in the context of diabetes. We focus on how inherited variation relates not only to hepatic fat accumulation but also to metabolic traits, cardiovascular disease and fibrosis. We go beyond variant spotting to explore evidence from genetic association studies, causal inference approaches and emerging translational research relevant to precision diabetes care.

## Genetic architecture of hepatic steatosis and MASLD

Twin and family studies indicate that genetic factors contribute substantially to variation in hepatic fat content and susceptibility to MASLD. Early studies using ultrasonography reported low heritability [7], but later investigations using MRI-proton density fat fraction (MRI-PDFF) estimated the heritability of steatosis and fibrosis to be approximately 50% [8]. This highlights the importance of accurate phenotyping.

Family-based studies provide convergent evidence [9]. First-degree relatives of individuals with MASLD-related cirrhosis have a significantly higher risk of advanced fibrosis [10, 11], and national registry data demonstrate familial clustering of cirrhosis, hepatocellular carcinoma and liver-related mortality [12]. At the genomic level, common variants captured by genome-wide association study (GWAS) arrays explain approximately 24–27% of variance in hepatic fat content, consistent with heritability estimates from twin studies and reflecting the aggregate contribution of genome-wide common variation rather than only currently identified GWAS loci [13, 14]. This suggests that a substantial proportion of MASLD risk is genetically mediated and identifiable through large-scale discovery efforts.

GWAS have interrogated MASLD using imaging-derived measures of hepatic steatosis, circulating liver enzymes, binary disease definitions from diagnostic codes and histological phenotypes. The first major locus identified was *PNPLA3* I148M, which is associated with hepatic triglyceride accumulation and with progressive stages of disease, including steatohepatitis [15], fibrosis and hepatocellular carcinoma [13, 16, 17]. Subsequent studies identified *TM6SF2* E167K, implicating impaired VLDL secretion in steatosis pathogenesis [18].

Computed tomography (CT)-based studies have replicated associations with MASLD for *PNPLA3* and *TM6SF2* and identified *PPP1R3B*, a locus related to hepatic glycogen metabolism rather than triglyceride accumulation [13]. Recent exome-wide analyses in the UK Biobank have also identified rare coding variants, including in *LMF2*, that contribute to hepatic steatosis burden [19]. Although CT-based studies enable large sample sizes, CT lacks sensitivity compared with MRI and may conflate visceral adiposity with hepatic steatosis, potentially introducing noise into genetic signal interpretation. Furthermore, the limited capacity of CT to differentiate hepatic triglyceride from glycogen deposition may complicate the interpretation of associations at loci such as *PPP1R3B,* which primarily impact glycogen metabolism.

More recent large-scale GWAS using MRI-PDFF have expanded the list of loci to include variants in or near *GCKR*, *APOE*, *TRIB1*, *GPAM*, *MTARC1* and *MBOAT7* [5, 20–24]. These loci map to pathways involving lipid droplet biology, hepatic lipogenesis, phospholipid remodelling and mitochondrial detoxification. Inclusion of non-European populations has revealed that, as with other complex traits, the effects and detectability of these loci are likely to vary across populations because of differences in allele frequencies and linkage disequilibrium structure [21]. Larger multi-ancestry studies will be needed to clarify whether population-specific signals exist.

Exome-wide association studies using imaging data have also explored the impact of rare variants (minor allele frequency of <0.1%) on liver fat content. A CT-based study identified a rare coding variant in *LMF2* that was positively associated with liver fat content as well as increased serum alkaline phosphatase, total cholesterol and BMI [19]. A study conducted in ~18,000 UK Biobank participants explored whether rare inactivating variants might impact liver fat according to MRI-derived liver fat measurements [25]. Rare inactivating variants in apolipoprotein B (*APOB)* and microsomal triglyceride transfer protein (*MTTP*), both known to have key roles in lipid homeostasis, were associated with increased liver fat. The rare variant in *APOB* was also associated with higher concentrations of aspartate aminotransferase (AST) and alanine aminotransferase (ALT) but lower circulating lipoprotein concentrations [25]. In another study, the *ATG7* rs143545741 C>T mutation, encoding the p.P426L amino acid substitution in autophagy-related 7, was found to be associated with an increased risk of MASLD [26]. Targeting re-sequencing analysis of *PNPLA3*, *TM6SF2* and *MTTP* identified a rare variant in *PNPLA3* that was positively associated with MASLD risk and a rare variant in *MTTP* that was negatively associated with risk [27]. A separate study identified a different rare variant in *MTTP* (rs745447480) that was associated with the development of severe MASLD in a four-generation family with no typical disease risk factors [28].

Beyond imaging, serum biomarkers such as ALT and composite indices such as the Fatty Liver Index have also been used in GWAS [20, 29–31]. These studies show partial overlap with imaging-based loci but also identify variants that are more closely related to hepatocellular stress or inflammation rather than fat accumulation per se. This suggests that liver enzyme elevation may reflect hepatocellular stress or inflammation, not just lipid accumulation.

Histology-based GWAS are considered the gold standard for identifying genetic associations with MASLD, but are costly and invasive and therefore remain relatively limited in scale. Studies of biopsy-confirmed MASLD have reported associations for variants in or near *PNPLA3*, *TM6SF2*, *HSD17B13* and *GCKR* across histological stages [32]. Imaging markers of inflammation and fibrosis, such as corrected T1 (cT1), are also associated with several MASLD loci, although many effects, including those of *PNPLA3* and *TM6SF2*, appear to be mediated by hepatic steatosis rather than reflecting direct fibrogenic pathways [22, 24]. The text box summarises reproducible MASLD loci that meet genome-wide significance (*p*<5 × 10^−8^) across imaging-based, histological and validated diagnostic phenotypes.

**Figure:**
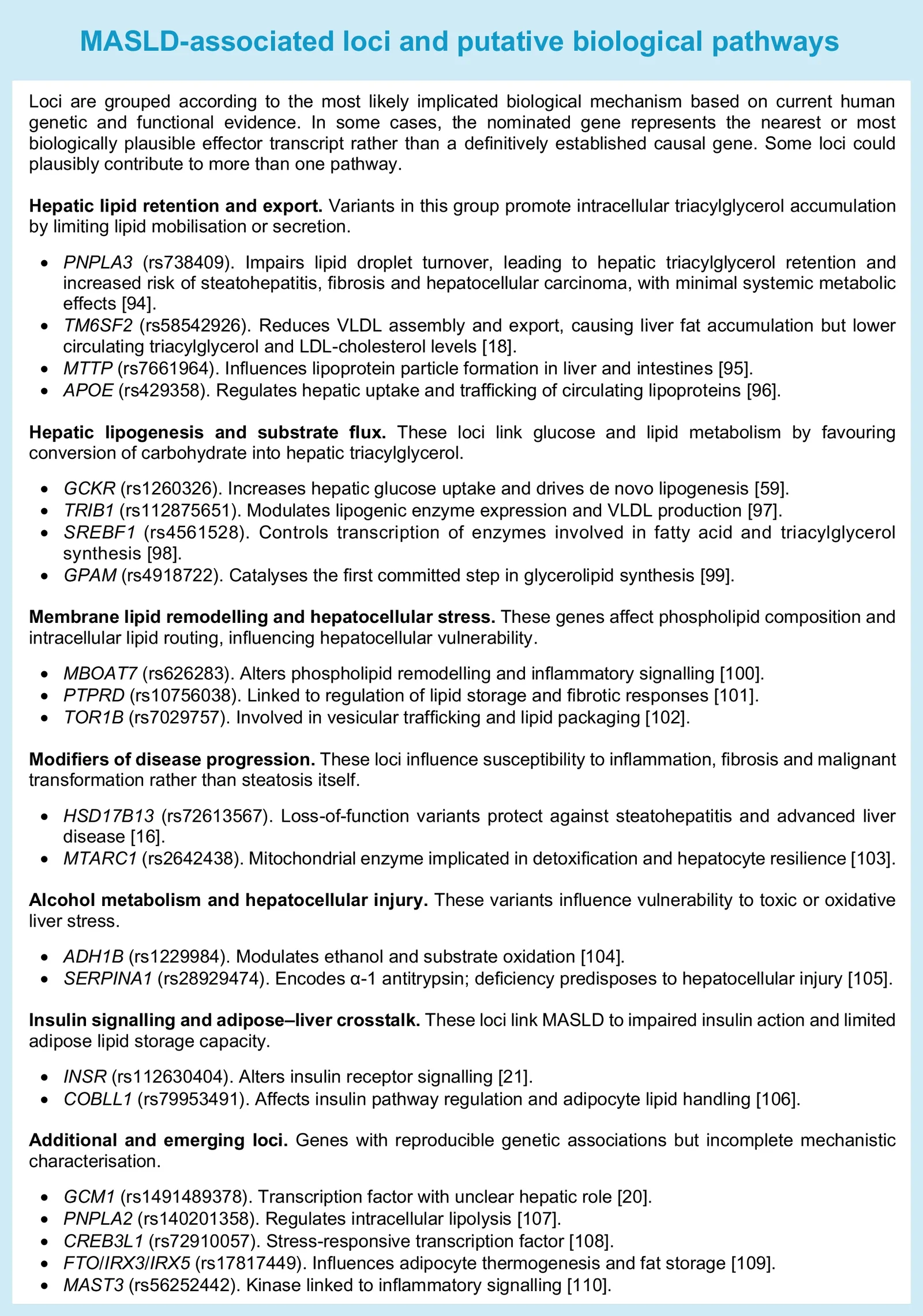

Integration of GWAS with epigenomic and transcriptomic data indicates that many MASLD variants localise to hepatocyte-specific regulatory regions, supporting liver-intrinsic mechanisms [24, 33, 34]. However, some loci, including *GCKR* and *TRIB1*, show activity across metabolic tissues and influence systemic traits such as insulin resistance and circulating lipids [35].

To date, the largest imaging-based GWAS studies of hepatic steatosis and related phenotypes identified 17 loci [21], although the precise number varies depending on phenotype definition, ancestry and study design. Recent imaging-based GWAS using MRI-derived liver fat measurements have typically included 30,000–65,000 participants [21, 36], whereas studies using biomarker-based proxies such as liver enzymes, composite indices or electronic-based health records have analysed cohorts of >100,000 individuals [31, 37]. As sample sizes increase and more diverse populations are included, it is likely that additional loci and biological pathways contributing to MASLD susceptibility will be identified, consistent with the highly polygenic architecture observed for other complex metabolic diseases.

## Genetic overlap and divergence between hepatic steatosis and systemic metabolism

Although MASLD commonly coexists with obesity and insulin resistance, genetic and metabolic studies indicate that hepatic steatosis can arise through biologically distinct routes. In many individuals, hepatic steatosis reflects systemic metabolic overload driven by insulin resistance, dyslipidaemia and excess substrate delivery. In others, steatosis appears to result primarily from hepatocyte-intrinsic defects in lipid handling, with relatively modest effects on whole-body metabolism. This distinction is specifically relevant in diabetes, where hepatic steatosis may represent either a manifestation of global metabolic dysfunction or a predominantly hepatic disorder that is metabolically ‘silent’.

**Fig. 1:**
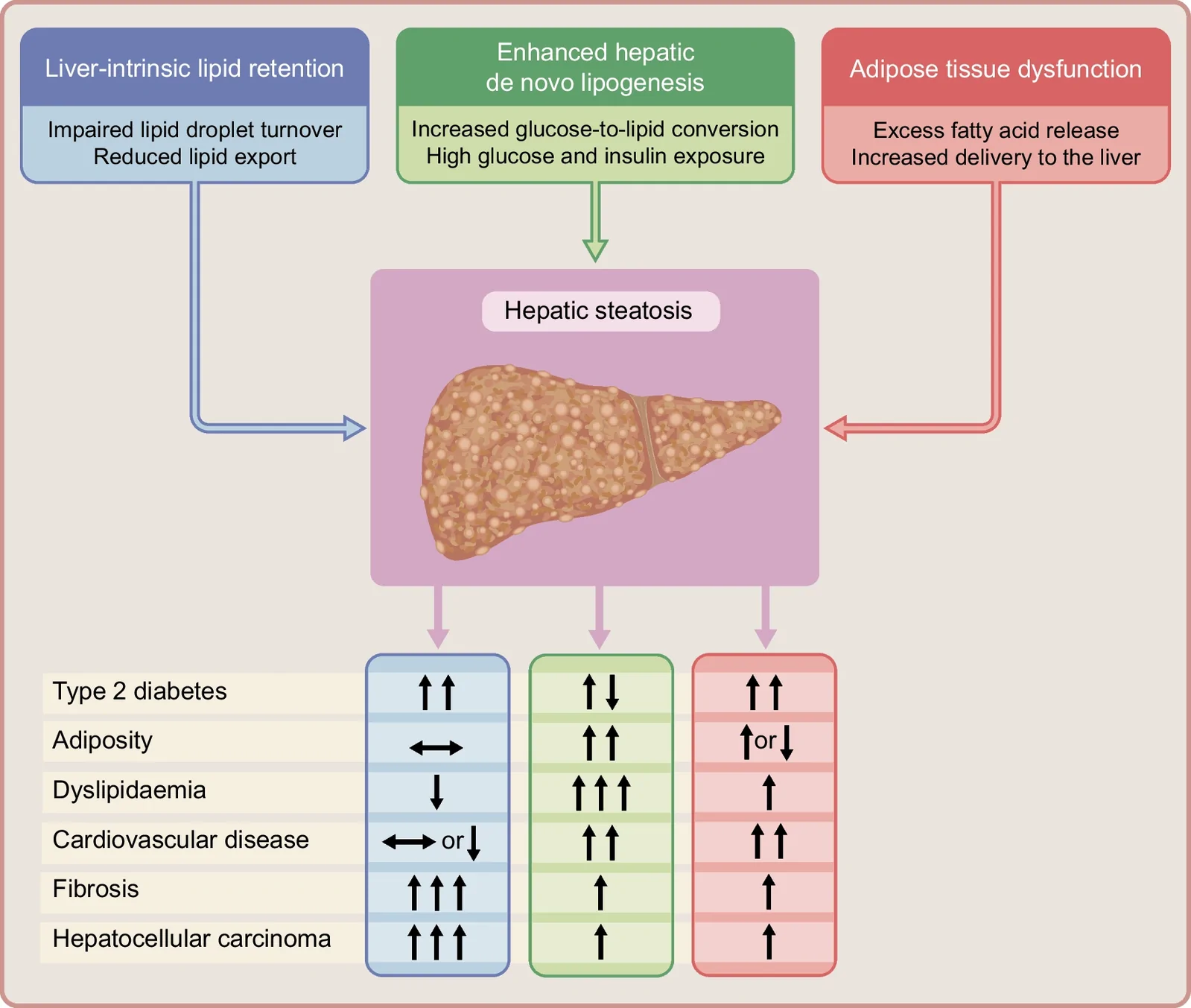
Distinct biological pathways leading to hepatic steatosis and their differential associations with metabolic and clinical outcomes. Hepatic steatosis can arise through multiple mechanisms, including (1) liver-intrinsic lipid retention; (2) enhanced hepatic de novo lipogenesis in the context of hyperglycaemia and hyperinsulinaemia; and (3) excessive fatty acid delivery from dysfunctional adipose tissue. Although these pathways converge on a shared phenotype of liver fat accumulation, they differ in their associations with type 2 diabetes, cardiovascular disease and liver-related outcomes. Variants such as *PNPLA3* and *TM6SF2* predominantly promote liver-intrinsic steatosis with strong associations with progressive liver disease but limited systemic metabolic effects. In contrast, variants linked to lipogenesis and adipose dysfunction are more closely associated with insulin resistance, type 2 diabetes and cardiovascular risk. This figure is available as part of a downloadable slideset

Physiological and genetic evidence indicates that MASLD can be driven by at least three dominant pathogenic processes: (1) liver-intrinsic lipid retention; (2) enhanced hepatic de novo lipogenesis in the context of hyperglycaemia and hyperinsulinaemia; and (3) excessive fatty acid delivery from dysfunctional adipose tissue [38] (Fig. 1). Independent network inference approaches converge on a similar tripartite architecture, reinforcing that hepatic steatosis is a shared phenotype arising from distinct upstream biological routes [39]. These mechanisms are not mutually exclusive but differ in their relative contributions between individuals, helping to explain why steatosis may be strongly linked to cardiometabolic risk in some people but is relatively uncoupled from it in others [38].

**Fig. 2:**
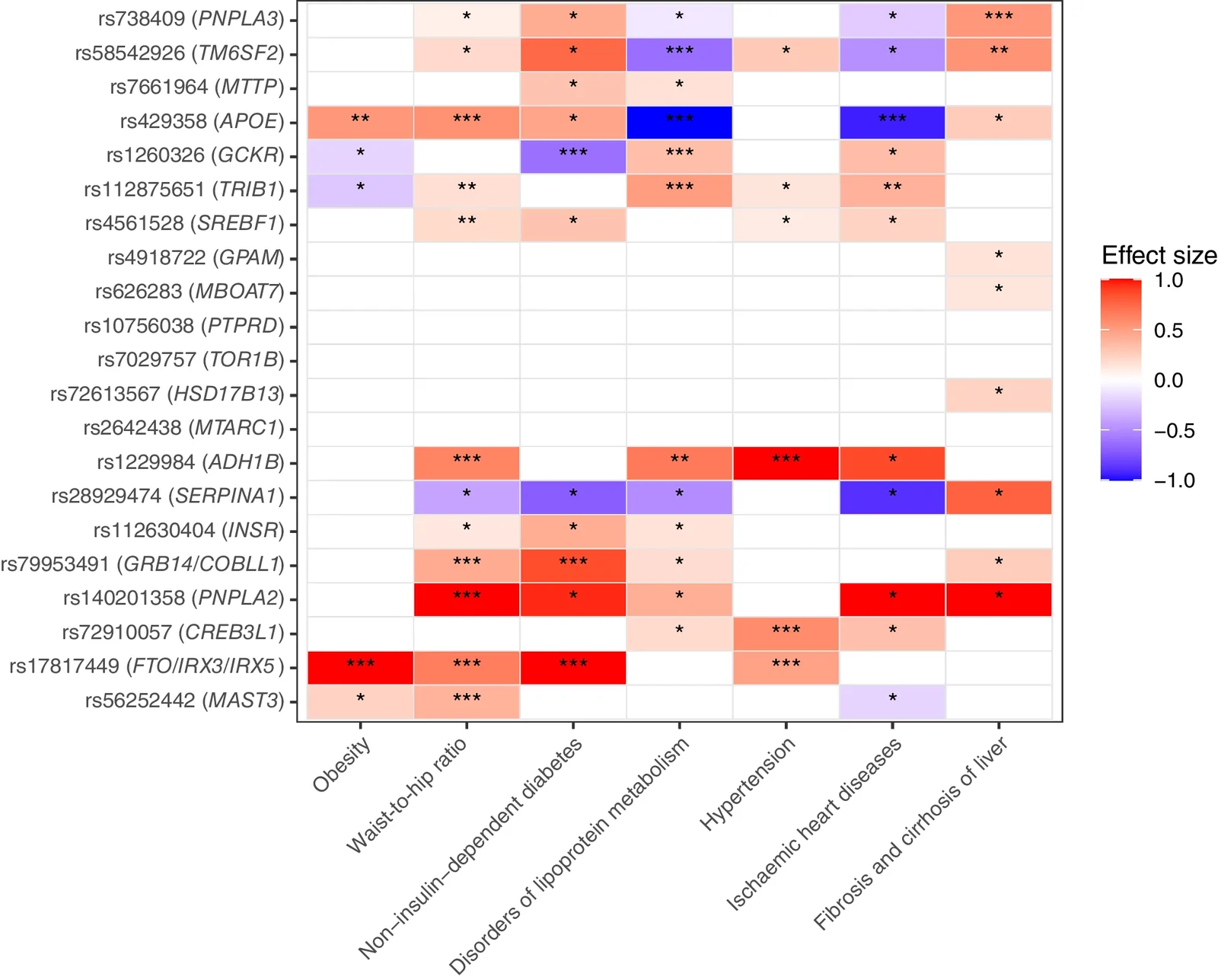
Effects of MASLD risk (or liver fat-increasing) alleles on cardiometabolic and liver-related traits derived from the Gene ATLAS web tool [111]. Rows represent the variants listed in the text box, labelled by rsID with the nearest or most likely candidate gene shown in parentheses. The *GCM1* variant (rs1491489378) was not available from the Gene ATLAS web tool and is therefore not included. Columns represent traits. Tile colour indicates the direction and magnitude of the effect estimate (β), with red denoting positive and blue denoting negative associations. Effect sizes are scaled within each trait to improve visual resolution while preserving the direction of effect. Colour intensity reflects relative effect sizes within each trait. Only nominally significant associations (*p*<0.05) are shaded. *p<0.05; **p<1 × 10^−^^5^; ***p<5 × 10^−8^. This figure is available as part of a downloadable slideset

GWAS loci map onto these broad pathogenic domains. Variants in loci such as *GCKR* and *TRIB1* are associated with increased hepatic steatosis together with higher triglyceride and LDL-cholesterol levels, increased risk of myocardial infarction and lower risk of type 2 diabetes, while showing relatively weak associations with cirrhosis and hepatocellular carcinoma [5] (Fig. 2). These loci influence hepatic glucose flux and de novo lipogenesis, embedding steatosis within a wider metabolic phenotype [5, 17]. By contrast, variants such as *PNPLA3* and *TM6SF2* produce large increases in hepatic triglyceride content and confer substantially higher risks of cirrhosis and hepatocellular carcinoma, while showing a favourable association with circulating lipids (lower triglycerides and LDL-cholesterol levels), reduced risk of myocardial infarction and higher risk of type 2 diabetes [5, 17] (Fig. 2). These variants act mainly through liver-intrinsic mechanisms, such as impaired lipid droplet turnover or reduced VLDL export [5, 6, 17, 35].

Variation in adipose tissue expandability provides specific insight into the third pathway driving MASLD: excessive fatty acid delivery from dysfunctional adipose tissue. The distinction between favourable and unfavourable adiposity demonstrates that obesity can arise through biologically different routes with divergent consequences for ectopic fat deposition [40–44]. Favourable adiposity variants, exemplified by *PPARG*, promote preferential expansion of subcutaneous fat depots while preserving insulin sensitivity and limiting lipid spillover to the liver, attenuating engagement of the adipose-driven pathway despite higher BMI [40–44]. By contrast, unfavourable adiposity variants, including those at the *FTO* locus, are characterised by impaired peripheral fat storage and increased lipid partitioning into visceral and hepatic compartments, promoting steatosis together with insulin resistance and elevated type 2 diabetes risk [40–44]. Monogenic obesity due to *MC4R* deficiency further supports the concept that adipose lipid partitioning determines metabolic risk. Individuals with *MC4R* deficiency present with severe obesity but reduced circulating lipid levels and enhanced triglyceride clearance, indicating efficient adipose lipid storage and reduced ectopic lipid delivery [45]. Thus, obesity does not necessarily imply adipose tissue dysfunction or uniform risk of MASLD.

Imaging genetics provides a spatial readout of these mechanistic differences by showing how distinct genetic pathways translate into different patterns of fat distribution across organs [46]. Variants that primarily act through liver-intrinsic lipid retention, such as *PNPLA3* and *TM6SF2*, disproportionately increase hepatic fat with relatively minimal effects on peripheral or visceral adipose depots, consistent with activation of a liver-dominant pathway of MASLD [46]. By contrast, variants influencing hepatic lipogenesis and systemic lipid partitioning present broader anatomical signatures. Genetic loci such as *FTO*, *ADH1B* and *APOE* are associated with higher fat across multiple depots, including abdominal and thigh subcutaneous adipose tissue, visceral fat and pancreatic fat, whereas liver fat-increasing alleles at loci including *GPAM*, *GCKR*, *TRIB1*, *COBLL1* and *PPARG* are associated with relatively lower peripheral subcutaneous fat, consistent with limited adipose storage capacity and preferential ectopic lipid deposition [46].

Clustering approaches that integrate clinical, metabolic and genetic data further reinforce this framework [5, 6, 47]. A ‘liver-specific’ cluster enriched for *PNPLA3* risk alleles shows rapid progression of liver disease but relatively low cardiovascular risk, whereas a ‘systemic’ cluster characterised by hyperglycaemia and hypertriglyceridaemia shows similar liver disease incidence but substantially higher cardiovascular and diabetes risk [5, 6, 47]. For people with diabetes, this heterogeneity may help explain why similar degrees of steatosis are associated with divergent risks of glycaemic deterioration, cardiovascular disease and fibrosis [17].

## Gene–diabetes interactions: the modifying role of hyperglycaemia, insulin resistance and lifestyle

Building on the distinction between liver-dominant and systemically driven pathways of MASLD, the expression of genetic susceptibility can be further modified by the metabolic environment in which it operates. Type 2 diabetes alters substrate availability, insulin signalling and adipose–liver lipid flux, which can influence how risk variants manifest phenotypically. Rather than acting as fixed determinants, many MASLD-associated genotypes show context-dependent effects that vary with hyperglycaemic exposure, insulin resistance and adiposity. This framework implies that genetic risk should be interpreted conditionally, rather than as a fixed predictor, because its phenotypic expression depends on metabolic state.

One pathway involves hepatic carbohydrate processing. Variants that increase glucose entry into hepatocytes, particularly those affecting glucokinase regulation such as *GCKR*, favour diversion of carbohydrates towards lipid synthesis. Persistent hyperglycaemia and hyperinsulinaemia, as seen in type 2 diabetes, may amplify this routing towards hepatic triglyceride accumulation [48]. Physiological modelling suggests that insulin secretion itself, rather than the circulating insulin concentration alone, may be particularly relevant to this pathway, as hepatic insulin clearance can obscure true beta cell output [39].

Even variants that primarily affect hepatocyte lipid handling appear sensitive to metabolic context. *PNPLA3* provides a clear example. In metabolically healthy individuals, its effect on hepatic steatosis is modest, whereas in obesity or diabetes the impact is substantially greater [49]. *PNPLA3*-associated risk also varies by age, sex and ancestry [49]. Similar interactions are observed for fibrosis severity and liver-related outcomes [50, 51].

Environmental and behavioural exposures represent an additional layer of modification. Physical activity and healthier dietary patterns are associated with weaker expression of inherited risk of MASLD [52, 53], whereas unfavourable diets, smoking and alcohol intake appear to strengthen genetic associations with hepatic steatosis and liver-related mortality [54–56]. These observations further support a model in which genetic effects are embedded within behavioural and metabolic contexts.

Gene–diabetes interactions therefore provide a mechanistic explanation for interindividual variability in disease course and treatment response. Potential therapeutic implications are discussed in the section on ‘Translational implications’.

## Causal inference: disentangling directionality between hepatic steatosis and diabetes

Mendelian randomisation provides a framework to test whether lifelong differences in hepatic steatosis influence diabetes risk [57]. However, the mechanistic heterogeneity outlined above has important implications for causal interpretation. Because hepatic steatosis can arise through liver-intrinsic lipid retention, substrate-driven lipogenesis or adipose tissue dysfunction, ‘genetically increased hepatic steatosis’ does not represent a single biological exposure. Network-based causal analyses similarly indicate that liver fat lies downstream of multiple upstream determinants [39]. Consequently, Mendelian randomisation analyses that aggregate genetic variants across mechanistically distinct loci may obscure pathway-specific causal effects. Cross-trait genetic analyses further support this interpretation, demonstrating a strong genome-wide genetic correlation between MASLD and type 2 diabetes (r_g_≈0.65) together with shared transcriptomic signatures across metabolically relevant tissues, indicating substantial polygenic overlap rather than a single shared pathway [35].

The validity of Mendelian randomisation in this context therefore depends critically on which variants are used as instruments. Most liver fat-increasing alleles are associated with a higher risk of type 2 diabetes, although different mechanisms confer different magnitudes of effect. For example, instruments enriched for liver-restricted mechanisms, such as *PNPLA3* and *TM6SF2*, exert particularly strong effects on diabetes susceptibility [5]. Consistent with this, bidirectional Mendelian randomisation analyses using these variants demonstrate that genetically driven hepatic steatosis can increase diabetes risk even in the absence of systemic insulin resistance, supporting a pathway mediated primarily through impaired insulin secretion rather than classical insulin resistance [58]. By contrast, variants linked to hepatic substrate use, including *GCKR* and *TRIB1*, display broader metabolic pleiotropy [5, 6]. These loci promote hepatic fat accumulation in parallel with reducing the risk of type 2 diabetes [5]. One plausible explanation is that these variants enhance hepatic conversion of glucose to fatty acids via de novo lipogenesis [59]. Distinguishing such mechanisms is therefore essential when interpreting causal estimates derived from genetic instruments.

Mendelian randomisation analyses also indicate the presence of asymmetrical bidirectionality. Genetic liability to insulin resistance and type 2 diabetes is associated with a higher risk of hepatic steatosis, consistent with the role of hyperinsulinaemia and excess substrate flux in steatosis [60], whereas cross-trait genetic analyses suggest that MASLD itself exerts a causal effect on diabetes risk, with stronger and more consistent estimates than those observed for the reverse direction [35]. Reciprocal effects have also been demonstrated, with genetic predisposition to hepatic steatosis increasing the risk of type 2 diabetes, and genetic risks of diabetes, adiposity and triglyceride accumulation increasing susceptibility to hepatic steatosis [58]. These findings support a feedback model in which metabolic dysfunction and hepatic steatosis amplify one another rather than acting as strictly linear causal traits.

A recent two-step Mendelian randomisation analysis using subtype-specific diabetes outcomes demonstrated that genetically predicted MASLD increases the risk of severe insulin-resistant, severe insulin-deficient and age-related diabetes subtypes, but not mild obesity-related diabetes, with liver fat explaining the largest proportion of mediation [61]. These results reinforce the concept that hepatic steatosis does not uniformly influence diabetes risk; instead, its diabetogenic effect depends on the metabolic and genetic context in which it arises. Importantly, circulating insulin concentrations reflect both secretion and hepatic clearance, and network-based analyses using C-peptide-derived insulin secretion estimates suggest that excessive beta cell output, rather than circulating insulin levels alone, may be the physiologically relevant driver of hepatic lipid accumulation [39].

Genome-wide overlap analyses further suggest that these causal structures partly reflect shared polygenic architecture, with multiple loci jointly influencing susceptibility to both hepatic steatosis and diabetes [35]. These observations indicate that asking whether ‘genetically driven hepatic steatosis causes diabetes’ is an oversimplification. Interpreting Mendelian randomisation results in MASLD therefore requires stratification by biological mechanism rather than aggregation across heterogeneous genetic instruments.

## Clinical consequences of genetic heterogeneity in MASLD

Genetic risk for MASLD does not stop at steatosis. It contributes to heterogeneity in the trajectory of liver disease and extrahepatic complications, particularly in people with diabetes. Individuals with similar clinical profiles, such as similar HbA_1c_ or BMI, can experience different liver outcomes ranging from minimal fibrosis to cirrhosis or hepatocellular carcinoma. As discussed in ‘Genetic overlap and divergence between hepatic steatosis and systemic metabolism’, MASLD can arise through biologically distinct pathways. These differences in underlying disease drivers help explain why similar degrees of liver fat can be associated with divergent clinical trajectories.

The strongest genetic signals for adverse hepatic outcomes involve variants that impair hepatic lipid handling and export: *PNPLA3* (I148M) and *TM6SF2* (E167K) [5]. These loci are consistently associated with increased risks of fibrosis, cirrhosis and hepatocellular carcinoma across MASLD cohorts. In populations with diabetes, their effects appear amplified and interact with metabolic burden and fibrosis stage to influence hepatic decompensation and liver-related mortality [62]. Among individuals with similar FIB-4 scores, some progress to cirrhosis or decompensation and others do not [63]. Genetic differences (e.g. *PNPLA3* and *TM6SF2*) are likely to contribute to this divergence and may help identify individuals who require closer follow-up, imaging or referral [63].

Genetic determinants of MASLD show divergent associations with cardiometabolic disease. Variants that predominantly drive liver injury, including *PNPLA3* and *TM6SF2*, reduce hepatic lipoprotein export and are associated with lower circulating LDL-cholesterol and triglyceride levels [5]. As a result, these variants show weak or inverse associations with cardiovascular events, despite strongly increasing liver-related risk. Community-based cohort studies of *TM6SF2* illustrate this dissociation, with opposing directions of effect for hepatic and cardiac outcomes [64].

By contrast, MASLD-associated variants linked to insulin signalling, glucose handling and de novo lipogenesis show stronger associations with systemic complications, including progression of type 2 diabetes, hypertension, heart failure, chronic kidney disease and all-cause mortality [5]. Polygenic clustering approaches consistently distinguish two broad biological subtypes: a liver-dominant genetic subtype driven primarily by hepatocellular lipid retention, and a systemic metabolic subtype in which hepatic steatosis reflects underlying insulin resistance and metabolic dysfunction [5, 6]. In people with diabetes, this framework helps explain why MASLD may behave either as a primarily hepatic disorder or as one component of a wider cardiometabolic syndrome phenotype.

Despite these mechanistic differences, genetically driven hepatic steatosis is consistently associated with increased risk of cirrhosis, hepatocellular carcinoma and intrahepatic bile duct and gallbladder cancers, irrespective of the underlying mechanism generating hepatic fat [5]. These associations follow a graded, dose-dependent pattern, such that greater liver fat burden confers a progressively higher risk [5].

## Translational implications

The genetic heterogeneity underlying MASLD in people with diabetes has direct translational implications for risk stratification, treatment response and therapeutic target discovery. If distinct genetic pathways lead to different clinical trajectories, genetics offers a means to move beyond uniform risk assessment towards mechanism-informed prediction and intervention. This concept underpins emerging precision medicine approaches in which inherited susceptibility is integrated with clinical phenotypes to refine prognostic assessment and guide therapeutic decision-making.

Polygenic risk scores summarise the cumulative contribution of multiple MASLD-associated variants into a single quantitative estimate of inherited susceptibility. In both general and diabetes-enriched populations, MASLD polygenic risk scores are associated with hepatic steatosis [65–68] and with more advanced phenotypes, including steatohepatitis [35, 69], fibrosis [69] and hepatocellular carcinoma [70, 71]. In people with type 2 diabetes, such scores may refine prognostic stratification by identifying individuals at particularly high genetic risk of progressive liver disease beyond that captured by routine clinical markers [72].

Beyond conventional polygenic risk scores, clustering and pathway-partitioned approaches aim to group genetic variants according to their underlying biological mechanisms. These methods may better capture the heterogeneity of MASLD by distinguishing between liver-intrinsic and systemic metabolic drivers of disease. In principle, such approaches could provide greater mechanistic resolution than single variants or aggregate genome-wide scores, particularly for stratifying individuals into biologically informed subgroups. However, evidence that these approaches improve predictive performance over individual high-impact variants or conventional polygenic scores remains limited, and their incremental clinical value requires further evaluation in prospective and comparative studies.

**Fig. 3:**
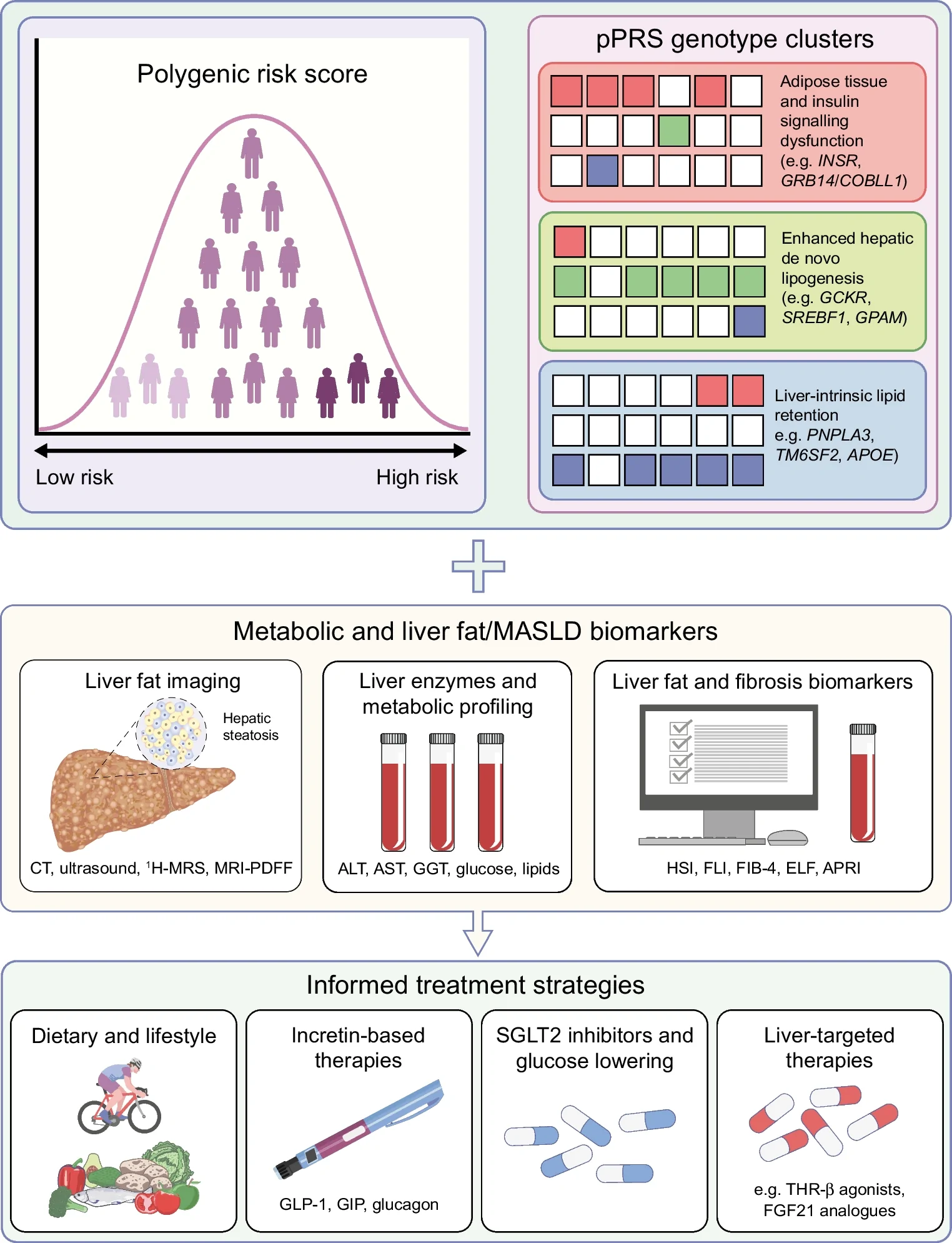
Mechanism-informed precision framework for MASLD risk stratification in people with diabetes. Partitioned polygenic risk scores (pPRS) capture inherited susceptibility across major biological pathways contributing to hepatic steatosis, including adipose tissue and insulin signalling dysfunction, enhanced hepatic de novo lipogenesis and liver-intrinsic lipid retention. Integration of pathway-specific genetic scores with clinical, metabolic and liver-specific biomarkers, such as liver fat imaging, circulating liver enzymes, cardiometabolic profiling and non-invasive fibrosis indices, may enable improved risk stratification and support mechanism-informed management strategies. In the schematic, each row in the ‘pPRS genotypes’ panel represents the presence or absence of risk alleles contributing to a specific pathway-defined score. For illustrative purposes, a single primary colour denotes the predominant pPRS pathway for each example individual. However, this representation is intentionally simplified. In clinical reality, most individuals will carry risk alleles across multiple pathways, and MASLD pathogenesis typically reflects overlapping rather than strictly dominant biological processes. APRI, AST-to-platelet ratio index; ELF, Enhanced Liver Fibrosis score; FIB-4, Fibrosis-4 index; FLI, Fatty Liver Index; FGF21, fibroblast growth factor 21; GGT, gamma-glutamyl transferase; GIP, glucose-dependent insulinotropic polypeptide; GLP-1, glucagon-like peptide-1; ^1^H-MRS, proton magnetic resonance spectroscopy; HSI, hepatic steatosis index; SGLT2, sodium–glucose cotransporter 2; THR-β, thyroid hormone receptor beta. This figure is available as part of a downloadable slideset

Figure 3 illustrates one potential framework for translating this genetic heterogeneity into clinically meaningful stratification. In this schematic, inherited risk is partitioned across major biological pathways contributing to hepatic steatosis, including adipose tissue dysfunction, enhanced hepatic lipogenesis and liver-intrinsic lipid retention. Importantly, these pathways should not be interpreted as mutually exclusive disease categories. Most individuals are likely to carry susceptibility alleles across several biological pathways simultaneously, such that MASLD arises from the combined influence of multiple mechanisms rather than a single dominant driver. This overlapping architecture is conceptually analogous to the ‘palette model’ proposed for type 2 diabetes, in which individuals show varying contributions from different pathogenic processes rather than belonging to discrete disease subtypes [73, 74]. Recognising these overlapping biological contributions may also help explain the variability in clinical trajectories, including differential risks of fibrosis progression, cirrhosis and hepatocellular carcinoma.

Several limitations currently restrict the clinical use of polygenic risk scores. First, polygenic risk scores explain only a small proportion of variance in hepatic steatosis. For example, a validated score incorporating 75 common variants accounts for approximately 2.5% of variance in MRI-derived hepatic steatosis [66]. The limited variance explained suggests that current polygenic scores are unlikely to have substantial standalone clinical utility. Whether they provide meaningful incremental predictive value beyond established clinical, biochemical and imaging measures remains uncertain and appears modest in unselected populations. Further work is therefore required to evaluate their contribution within integrated prediction models and prospective clinical cohorts. Second, most MASLD GWAS have been conducted in populations of European ancestry [75], resulting in reduced performance in other populations and limiting global applicability. Third, polygenic risk scores do not incorporate environmental and behavioural modifiers, such as diet, alcohol intake, physical activity levels and glycaemic management, which can substantially amplify or attenuate genetic risk [75], as discussed in ‘Gene–diabetes interactions’. These considerations favour the development of multivariable risk models that embed genetic information within longitudinal clinical and biomarker data, rather than relying on genetic scores as standalone tools. Integrative probabilistic modelling combining hormonal, adiposity and proteomic variables demonstrates that combining markers from multiple biological domains substantially improves prediction of hepatic steatosis compared with single-domain approaches [39].

The incremental predictive value of polygenic risk scores over standard fibrosis tools, such as FIB-4 or AST-to-platelet ratio index (APRI), remains modest in unselected populations [76]. However, emerging evidence suggests that genetic information may be more informative in high-risk groups, including individuals with type 2 diabetes or obesity [72]. In these settings, polygenic scores and single high-impact variants, mainly *PNPLA3* I148M, may help stratify residual risk among individuals with intermediate clinical scores and help prioritise individuals for imaging or specialist referral [77]. These data favour integrated risk models combining genetics with clinical, biochemical and imaging measures.

Genetic heterogeneity may also contribute to variation in treatment response. The *PNPLA3* I148M variant is associated with increased MASLD severity and with greater reductions in hepatic steatosis following lifestyle intervention and bariatric surgery, which is likely to reflect the higher baseline hepatic lipid burden [78–80]. Some studies have suggested that hepatic responses to glucagon-like peptide-1 (GLP-1) receptor agonists are attenuated in *PNPLA3* I148M carriers, although this finding requires confirmation in larger, genotype-stratified trials [81]. Variation in genes involved in hepatic glucose handling, such as *GCKR*, may influence response to dietary interventions or therapies targeting hepatic glucose metabolism, but this has not been systematically evaluated in MASLD-specific cohorts [82]. Robust genotype-stratified data from pharmacological trials remain limited. Early pharmacogenetic studies, including those linking *CYP2C8* polymorphisms to differential responses to pioglitazone, highlight the potential relevance of genetics for future trial design [83]. These preliminary data point towards the potential of genotype-informed treatment algorithms for MASLD in type 2 diabetes, but widespread implementation will require systematic evaluation in stratified trials.

Studies in human genetics have also identified pathways that appear protective against progressive liver disease, offering potential therapeutic targets. Loss-of-function variants in *HSD17B13* (rs72613567) and destabilising variants in *MTARC1* are associated with lower risks of steatohepatitis, fibrosis and hepatocellular carcinoma without adverse effects on insulin sensitivity or lipids [16, 84]. Similarly, protective alleles in genes such as *CIDEB* further implicate hepatocyte-intrinsic lipid handling as a modifiable pathway [85].

Translational programmes based on these mechanistic insights are now emerging. In animal models, *PNPLA3* antisense oligonucleotides reverse steatohepatitis and fibrosis, and early-phase clinical trials of the *PNPLA3* antisense oligonucleotide AZD2693 have demonstrated safety and biological activity in *PNPLA3* I148M homozygotes [86, 87]. A Phase IIb trial of AZD2693 has recently been completed in this genotype-defined population (NCT05809934); however, as of April 2026, the findings of this study have not been published. RNA-based therapies targeting *HSD17B13*, such as rapirosiran, have shown robust hepatic knockdown with favourable early safety profiles [88, 89]. Although *TM6SF2* has not yet been directly targeted, downstream pathways and indirect modulators, including lysophosphatidic acid signalling and acetyl-CoA carboxylase inhibitors, have shown therapeutic potential in experimental models [68, 90].

These therapeutic leads have been guided not only by GWAS but also by integrative omics approaches. Recent studies combining transcriptomic, proteomic and single-cell sequencing have provided granular insights into disease mechanisms and identified cell-specific targets across hepatocytes, stellate cells and macrophage populations [91, 92]. These approaches have the potential to transform therapeutic discovery in MASLD by providing a roadmap that connects genetic variation to cellular dysfunction and clinical phenotypes.

## Future directions and research gaps

Most existing evidence on gene–environment and gene–drug interactions in MASLD are concentrated on a small number of loci, particularly *PNPLA3*, highlighting the narrow mechanistic scope of current translational genetics. Despite significant progress in delineating the genetic architecture of MASLD, current evidence remains disproportionately skewed towards discovery-phase analyses in European-ancestry populations, often drawn from general cohorts without explicit representation of individuals with type 2 diabetes. This lack of diversity limits transferability and clinical relevance in diabetes care [75]. To move towards actionable genetic insights in MASLD, especially within diabetes care, there is a pressing need for large-scale, multi-ancestry GWAS and meta-analyses explicitly powered to evaluate variant effects across diverse ancestries and sex and age strata in people with diabetes.

Equally critical is the need for improved MASLD phenotyping in diabetes populations. Much of the current literature relies on imprecise surrogate markers (e.g. elevated liver enzymes) or crude binary disease definitions from diagnostic codes. Such approaches obscure disease heterogeneity and weaken genotype–phenotype resolution. Future work should aim to integrate quantitative imaging modalities such as MRI-PDFF, cT1 and liver elastography alongside histological confirmation where feasible. The combination of imaging, validated biomarkers (e.g. FIB-4, Enhanced Liver Fibrosis [ELF] score) and multi-omic data (e.g. transcriptomic, proteomic and metabolomic profiles) could enable more precise classification of disease stage, biological subtype and disease trajectory. Network-based analytical approaches that integrate these data layers may be particularly valuable for distinguishing causal from consequential biological processes. Importantly, longitudinal designs are essential to distinguish genetic contributions to MASLD onset from those influencing progression, regression and extrahepatic complications.

Several underexplored areas warrant attention. First, MASLD genetics in people with type 1 diabetes has received little investigation. Given the unique metabolic context of type 1 diabetes, characterised by insulin deficiency, exogenous insulin therapy and longer disease duration, the interplay between genetic risk and hepatic outcomes is likely to diverge significantly from that observed in type 2 diabetes [93]. Second, the intersection of MASLD genetics and women’s health also remains underexamined. Life stages and hormonal states such as polyendocrine metabolic ovarian syndrome (previously known as polycystic ovary syndrome), pregnancy and menopause may uncover gene–hormone interactions that modify hepatic and metabolic disease trajectories. Third, the genetic architecture of MASLD in early-onset type 2 diabetes and adolescence also requires investigation. Early disease manifestation may reflect a greater genetic load and predict a more aggressive disease course. Fourth, the role of genetics in insulin resistance, as a crucial factor preceding the development of overt metabolic diseases, warrants further consideration, as it may provide insights into disease trajectory and progression. In parallel, the field must extend beyond discovery towards implementation science. Gene–environment and gene–treatment interactions, particularly responses to lifestyle interventions, pharmacotherapy (e.g. GLP-1 receptor agonists, sodium–glucose cotransporter 2 [SGLT2] inhibitors) and emerging MASLD-specific agents, are critical but remain in early stages of investigation. Embedding genetic analyses into prospective interventional trials will be necessary to define treatment effect heterogeneity and enable the development of stratified care pathways based on genetic risk or mechanism of disease.

Finally, before genetic testing is embedded in clinical care, real-world evaluation of its cost-effectiveness, feasibility and ethical acceptability is required. Questions remain around clinical interpretation, genetic counselling infrastructure, patient education and equitable access. Without these safeguards, precision hepatology in diabetes may risk becoming a niche offering rather than a transformative model of care.

## Conclusion

MASLD is highly prevalent in people with diabetes but the clinical course is heterogeneous. Human genetic studies have shown that hepatic fat accumulation can arise through distinct biological pathways with different consequences for glycaemic management, cardiometabolic risk and liver disease progression. Variants such as *PNPLA3* and *TM6SF2* mainly affect liver-specific steatosis with limited systemic effects, whereas variants influencing lipogenesis and insulin resistance, such as *GCKR* and *TRIB1*, link hepatic fat to broader metabolic disease.

Collectively, these insights refine causal understanding, help explain clinical heterogeneity and highlight genetically supported therapeutic targets. Future research should prioritise ancestry-inclusive genetic discovery, improved phenotyping in populations with diabetes, and integration of genetic analyses into MASLD clinical trials. Embedding genetic information into diabetes care pathways may support a route towards mechanism-based, precision management of MASLD in people with diabetes.

## Supplementary Information

Below is the link to the electronic supplementary material.

Slideset of figures (PPTX 482 KB)

## Abbreviations

ALT: Alanine aminotransferase
AST: Aspartate aminotransferase
CT: Computed tomography
cT1: Corrected T1
FIB-4: Fibrosis-4 index
GLP-1: Glucagon-like peptide-1
GWAS: Genome-wide association study
MASLD: Metabolic dysfunction-associated steatotic liver disease
MRI-PDFF: MRI-proton density fat fraction
NAFLD: Non-alcoholic fatty liver disease
